# *In situ* diagnostics of the crystal-growth process through neutron imaging: application to scintillators

**DOI:** 10.1107/S1600576716004350

**Published:** 2016-04-12

**Authors:** Anton S. Tremsin, Małgorzata G. Makowska, Didier Perrodin, Tetiana Shalapska, Ivan V. Khodyuk, Pavel Trtik, Pierre Boillat, Sven C. Vogel, Adrian S. Losko, Markus Strobl, L. Theil Kuhn, Gregory A. Bizarri, Edith D. Bourret-Courchesne

**Affiliations:** a Space Sciences Laboratory, University of California at Berkeley, Berkeley, CA 94720, USA; b Department of Energy Conversion and Storage, Technical University of Denmark, Frederiksborgvej 399, Roskilde 4000, Denmark; c European Spallation Source ESS AB, PO Box 176, Lund SE-221 00, Sweden; d Lawrence Berkeley National Laboratory, 1 Cyclotron Road, Berkeley, CA 94720, USA; e Paul Scherrer Institute, 5232 Villigen PSI, Switzerland; f Los Alamos National Laboratory, Los Alamos, NM 87545, USA

**Keywords:** neutron imaging, non-destructive testing, crystal growth, scintillators, *in situ* diagnostics

## Abstract

Neutrons are known to be unique probes in situations where other types of radiation fail to penetrate samples and their surrounding structures. In this paper it is demonstrated how thermal and cold neutron radiography can provide time-resolved imaging of materials while they are being processed (*e.g.* while growing single crystals). The processing equipment, in this case furnaces, and the scintillator materials are opaque to conventional X-ray interrogation techniques. The distribution of the europium activator within a BaBrCl:Eu scintillator (0.1 and 0.5% nominal doping concentrations per mole) is studied *in situ* during the melting and solidification processes with a temporal resolution of 5–7 s. The strong tendency of the Eu dopant to segregate during the solidification process is observed in repeated cycles, with Eu forming clusters on multiple length scales (only for clusters larger than ∼50 µm, as limited by the resolution of the present experiments). It is also demonstrated that the dopant concentration can be quantified even for very low concentration levels (∼0.1%) in 10 mm thick samples. The interface between the solid and liquid phases can also be imaged, provided there is a sufficient change in concentration of one of the elements with a sufficient neutron attenuation cross section. Tomographic imaging of the BaBrCl:0.1%Eu sample reveals a strong correlation between crystal fractures and Eu-deficient clusters. The results of these experiments demonstrate the unique capabilities of neutron imaging for *in situ* diagnostics and the optimization of crystal-growth procedures.

## Abstract 2

The unique possibilities enabled by neutron imaging for *in situ* remote diagnostics of microstructural characteristics during crystal growth are demonstrated, even when the materials and surrounding structures are opaque to other more conventional interrogation techniques. Neutron radiography is implemented to image remotely the uniformity of elemental distribution (*e.g.* dopant concentration) during crystal growth, the location of the liquid/solid interface and the presence of macroscopic crystal defects (*e.g.* cracks), all with a temporal resolution of 5–7 s.

## 1. Introduction   

While multiple novel scintillation materials have been discovered during the past decade (Cherepy *et al.*, 2008 ▸; Derenzo *et al.*, 2003 ▸; Combes *et al.*, 1999 ▸; Bourret-Courchesne *et al.*, 2009 ▸, 2010 ▸, 2012 ▸; Yang *et al.*, 2011 ▸; Tyagi *et al.*, 2013 ▸; Nikl & Yoshikawa, 2015 ▸), only a couple of them have made a successful transition to commercialization. This transition is extremely time consuming and usually requires many years of development in order to optimize the raw material purification process and crystal-growth procedures to the industrial scale. One of the main challenges is to maintain the fragile equilibrium between two conflicting requirements, preserving the scintillation performance of large crystals and increasing the yield of production. The former is governed by the complex interplay between energy-conversion mechanisms and material composition that naturally encompasses the lattice and dopant constituents, and also by the extrinsic defects inherent in the thermodynamics and chemistry of real crystals. Controlling, or at least accounting for, this large compositional variation on an industrial scale while targeting commercially viable production has been a serious challenge which has slowed the development of the next generation of scintillator materials. These must be obtained reproducibly in high yield, with a relatively large size and at a low cost of production.

To facilitate the rapid transition from research to commercial-sized crystals, a knowledge of the microstructure of these crystals and real-time imaging of defect formation during crystal growth can be used to guide the optimization of the manufacturing process and the improvement of γ- and X-ray detection in general. While multiple characterization techniques can be employed for post-manufacturing characterization of scintillation materials, both destructive and non-destructive, the long delay for feedback information makes post-processing studies not ideal for the rapid optimization and scale-up of crystal-growth processes. *In situ* techniques are very attractive in this respect as they can provide the basis for real-time feedback control of the crystal-growth parameters, such as furnace temperature profile, crystal pull speed, mosaicity, stoichiometry of elemental composition, presence of high strain fields within the crystal leading to its subsequent fracture *etc*. For the Bridgman–Stockbarger technique there are no methods that can ‘see’ through furnaces and sample containers to image the process (*e.g.* ampules prevent reliable characterization by acoustic methods). Thus far, researchers have had to rely on simulations of the process (*e.g.* Brandon & Derby, 1992 ▸; Sonda *et al.*, 2005 ▸) that can only be validated indirectly. Obviously, the methods for *in situ* characterization should be non-destructive, capable of remote sensing and of sufficient temporal resolution. Such techniques should also take into account the high temperatures needed for the growth of most materials and the fact that any sensing element placed in direct contact with the growth material can affect the growth process itself (*e.g.* change the uniformity of the temperature profile in the growth volume).

Since γ- and X-ray scintillators are built to efficiently absorb high-energy photons, the conventional X- and γ-ray interrogation techniques cannot be easily implemented for *in situ* studies. Moreover, the furnace structures required for synthesis, often built from metals, are opaque to photons as well. The ability of neutrons to penetrate many materials opaque to other more conventional types of radiation makes them very attractive for the *in situ* study of scintillator materials during their growth. Conventional white-spectrum neutron radiography can be used to image the presence of defects such as cracks, voids or impurities and is sometimes used to image the uniformity of the elemental composition, utilizing the difference in the attenuation coefficient for neutrons of various elements (Kardjilov *et al.*, 2011 ▸). In addition, energy-resolved neutron imaging offers a unique possibility to study some microstructural properties of crystals enabled by the measured transmission spectra for each pixel of a data set (Santisteban *et al.*, 2001 ▸; Kockelmann *et al.*, 2007 ▸; Tremsin *et al.*, 2013*a* ▸,*b* ▸, 2014 ▸). The elemental composition and distribution of temperature within the scintillator material can also be studied *in situ* through neutron resonance absorption from the depth and Doppler broadening of the resonances, respectively, provided neutron transmission spectra can be measured in each pixel of an image in the epithermal range of neutron energies (Mayers *et al.*, 1989 ▸; Stone *et al.*, 2005 ▸; Yuan *et al.*, 2005 ▸; Schillebeeckx *et al.*, 2012 ▸; Kai *et al.*, 2013 ▸; Tremsin *et al.*, 2013*a* ▸,*b* ▸, 2014 ▸; Tremsin, Kockelmann *et al.*, 2015 ▸; Festa *et al.*, 2015 ▸).

In this paper we demonstrate the possibility of *in situ* remote mapping of elemental composition, as well as imaging of cracks and voids, within a scintillator material kept at elevated temperatures during its growth. Moreover, it is demonstrated that the dynamics of elemental distribution and defect formation can be studied in real time (on a scale of several seconds, fast enough for the slow process of crystal growth). In particular, the uniformity of 0.1 and 0.5% Eu doping in a BaBrCl:Eu scintillator is imaged in our experiments with ∼5–7 s resolution, while the material is subject to multiple partial melting/solidification cycles. The interface between the solid and liquid phases is also observed by the change in the elemental composition in the liquid phase where the dopant distribution changes relative to the as-prepared charge. The tomographic reconstruction, performed *ex situ* in our experiment, also demonstrates the possibility of accurately investigating the three-dimensional distribution of elements, voids and cracks within the sample, with a sensitivity to structures above ∼50 µm in size.

## 2. Experimental setup   

**Figure 1:**
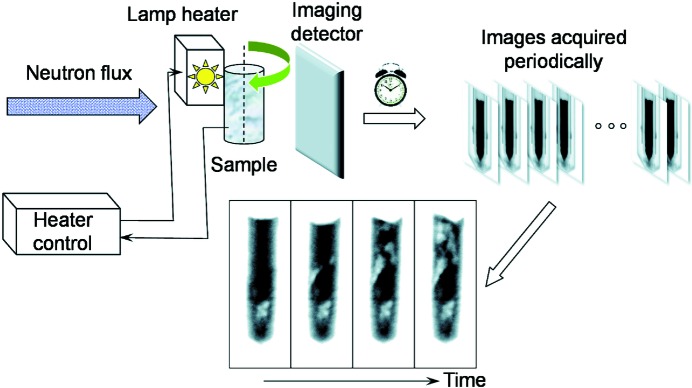
A schematic diagram of the experimental setup. The sample is heated from one side by a quartz lamp heater. White-spectrum neutron transmission images are recorded at regular intervals, every ∼4.7 or 6.7 s. The dynamics of Eu concentration are visualized in the sequence of images acquired after the heater temperature is changed to either ∼50 K above or below the melting point for heating and cooling cycles, respectively.

**Figure 2:**
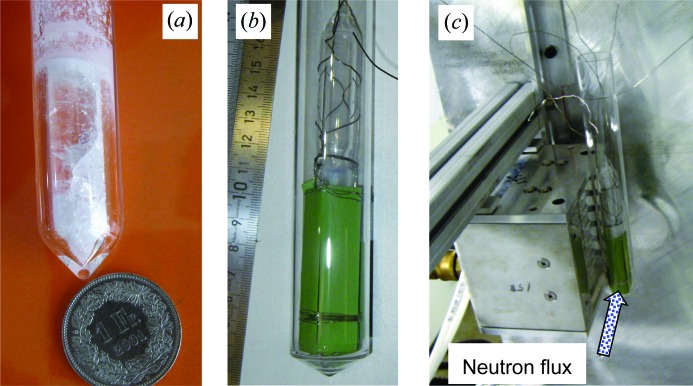
Photographs of the sample and the IR heater. (*a*) The BaBrCl:0.5% Eu sample sealed in a silica glass tube. (*b*) The sample surrounded by the NiO–YSZ light-absorber plates and suspended in a larger silica glass tube to prevent spills in case the sealed tube fractures. (*c*) The quartz lamp heater with the sample installed right next to it. The bottom of the sample was not heated by the lamp, in order to provide a solid–liquid interface during the measurements.

The experiments reported in this paper were conducted on two neutron imaging beamlines (BOA and ICON, providing a cold neutron spectrum) of the Paul Scherrer Institute, Villigen, Switzerland (Kaestner *et al.*, 2011 ▸; Morgano *et al.*, 2014 ▸; Tremsin, Morgano *et al.*, 2015 ▸). Fig. 1 ▸ shows a schematic diagram of the experimental setup: a parallel beam of neutrons (with *L*/*D* ratios of ∼280:1 to 350:1) illuminates the sample installed in front of a neutron imaging detector, integrating images over 4 or 6 s and saving them periodically at 4.7 and 6.7 s intervals using the CCD imaging cameras combined with ^6^Li/ZnS scintillator screens (Kaestner *et al.*, 2011 ▸). Thus, a set of transmission images *I*_sample_ was acquired in each experiment. These images were normalized by the image taken with no sample mounted in the beam, *I*_open_ (‘open-beam’ normalization) and corrected for detector dark noise *I*_dark_ (measured with the neutron beam blocked by the shutter). Thus, all the images shown in this paper are normalized according to the equation
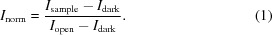
The BaBrCl:Eu studied in these experiments can be solidified as a solid solution without phase separation between BaBr_2_ and BaCl_2_. These solid solutions crystallize in an ortho­rhombic PbCl_2_ structure type (Gundiah *et al.*, 2013 ▸). The samples used in the present study were pre-synthesized as BaBrCl doped with Eu in polycrystalline form (millimetre-sized grains but not single crystals), and the orthorhombic structure was verified by X-ray diffraction. The samples were sealed in an evacuated silica glass tube with a 10 mm internal diameter (Fig. 2 ▸ *a*) because of the sensitivity of this material to moisture. The samples were mounted in the proximity of a quartz halogen lamp infrared heater maintaining a pre-set constant temperature at the thermocouple mounted on the exterior of the sample container. Therefore, the temperature of the ampule exterior was monitored in our experiments and not the precise temperature of the scintillation material itself, as in many real growth systems where the temperature is measured only at the perimeter of the heated region. The bottom part of the sample was intentionally not heated as it was installed below the heated zone in order to provide a temperature gradient within the sample, thus ensuring the presence of a solid/liquid interface in the sample. Therefore, the bottom part of the sample was never melted in our experiments and remained in as-prepared condition (polycrystalline charge). In order to reach the melting point of BaBrCl (∼1159 K), a sufficient amount of heat generated by the quartz halogen lamps needed to be absorbed by the sample. The absorption of light by the sample itself can change with temperature. It can also change after a phase transition. Therefore, a set of light absorbers (heat absorbers) was installed around the ampule in each experiment to change the heat-transfer mechanism from radiative to conductive, thus eliminating the dependence of the heat transfer on the sample phase or temperature. The heat absorbers were thin pieces of NiO–YSZ [yttria (Y_2_O_3_)-stabilized zirconia (ZrO_2_)] of much higher melting temperature than the measured BaBrCl samples. An Alumel (95% nickel, 2% manganese, 2% aluminium and 1% silicon) wire of ∼0.5 mm thickness was used to fix the heat absorbers around the silica glass ampule (Fig. 2 ▸ *b*). A water-cooled aluminium wall was installed in front of the detector to insulate it from the heat zone. The furnace itself was not custom-built for the present experiment but could be adapted for these proof-of-principle measurements. More details on the furnace structure can be found in the work of Makowska *et al.* (2015 ▸).

We must note that the lamp heater provides a strongly non-symmetrical heating environment, leading to strong convection in the melt and to a non-homogeneous melting and solidification front. During the rapid cooling process the solidification propagated primarily from top to bottom of the sample, as can be judged by the expulsion of Eu from the liquid phase, sometimes creating solidified clusters within the liquid phase. This is far from optimal for crystal growth and it was expected that polycrystalline samples would be formed, with strong segregation of the dopant in some areas. However, these proof-of-principle experiments allowed us to demonstrate the capabilities of the method. A dedicated Bridgman-type furnace for *in situ* crystal growth experiments is currently being designed and built.

**Figure 3:**
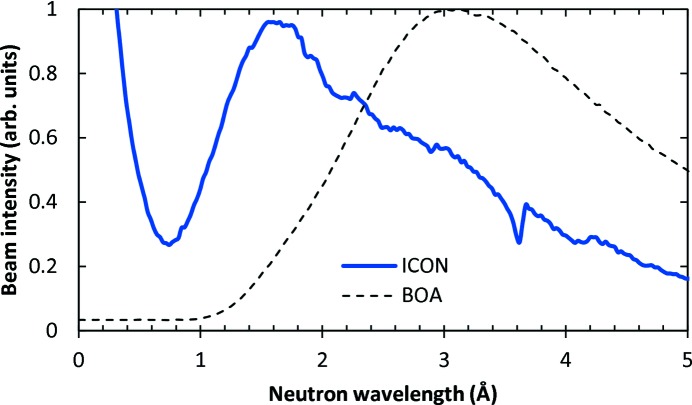
Neutron beam spectra for the ICON and BOA facilities where the experiments were conducted. The spectra are normalized to unit peak intensity. The cold neutron BOA beamline has a waveguide between the source and the sample position, which diverts the beam from the path of high-energy neutrons and γ-rays. Only neutrons with wavelengths above ∼1 Å are present in the beam. The ICON facility has some contribution from epithermal neutrons.

The neutron spectra of the two beamline facilities are shown in Fig. 3 ▸. *In situ* imaging while the samples were melted/solidified was performed on the BOA cold neutron beamline, where the neutron intensity peaks at ∼3 Å and there is no contribution of neutrons below 1 Å (Morgano *et al.*, 2014 ▸; Tremsin, Morgano *et al.*, 2015 ▸). The BOA beamline has a curved guide, which diverts neutrons from the direct view on the source, and thus nearly all the epithermal neutrons and γ-photons are excluded from the sample interrogation. Tomographic *ex situ* imaging of one sample after the last solidification cycle was performed on the ICON beamline (Kaestner *et al.*, 2011 ▸), which has a slightly warmer neutron spectrum and some contribution of epithermal neutrons. Only a very limited γ-ray contribution is present at this facility.

The tomographic data set consisted of 376 images, acquired over 360° (with 0.96° rotation between two consecutive projections), each integrated over a 60 s period. The initial image processing was performed by the *ImageJ* image-processing program (http://imagej.nih.gov/ij/). Reconstruction was performed by the *Octopus* reconstruction software (https://octopusimaging.eu/) and three-dimensional visualization performed by the *Avizo* three-dimensional analysis software (http://www.fei.com/software/avizo3d/).

### 2.1. Sample preparation   

Two BaBrCl:Eu samples were studied in our experiments.

#### 2.1.1. Sample N1   

A polycrystalline charge of 10 g of BaBrCl with a nominal concentration of 0.1 mol% Eu. The charge of raw materials was homogenized in a vacuum-sealed silica glass ampule by holding it at 1193 K (the melting point is about 1159 K) for 3 h. The subsequent cooling rate was about 60 K h^−1^. The charge consisted of millimetre-sized grains with no coloration from impurities.

#### 2.1.2. Sample N2   

A polycrystalline charge of 10 g of BaBrCl with a nominal concentration of 0.5 mol% Eu. The charge of raw materials was homogenized in a vacuum-sealed silica glass ampule by holding it at 1193 K (the melting point is about 1159 K) for 4 h. The subsequent cooling was directional at a translation rate of 1.2 mm h^−1^, followed by cooling to room temperature at about 40 K h^−1^. The charge consisted mainly of centimetre-sized grains with no coloration from impurities.

## 3. Results and discussion   

### 3.1. Theoretical neutron transmission   

Despite the relatively high penetration of neutrons for many γ-ray scintillation materials, obviously not all of them can be studied by white-beam neutron imaging for either of two reasons:

(i) Some specific elements, such as Gd, Cd, ^10^B and ^6^Li, are strong neutron absorbers and can therefore only be studied either with thin samples or when these elements or isotopes have a low concentration within the material composition.

(ii) The attenuation of neutrons for the elements present in the sample may be too low, such that no contrast in the images is observed from variation of the elemental composition or the presence of defects.

We have implemented a relatively simple model which allows the prediction of sample transmission for a given geometry and elemental composition. The model uses the tabulated energy-dependent attenuation cross sections for various elements/isotopes [Evaluated Nuclear Data File (ENDF), https://www-nds.iaea.org/exfor/endf.htm] and allows the calculation of expected sample transmission as a function of neutron wavelength or energy. The predicted spectra allow the estimation of sensitivity limits to a certain element concentration, as well as evaluation of the best beamline facility where a particular material can be investigated.

**Figure 4:**
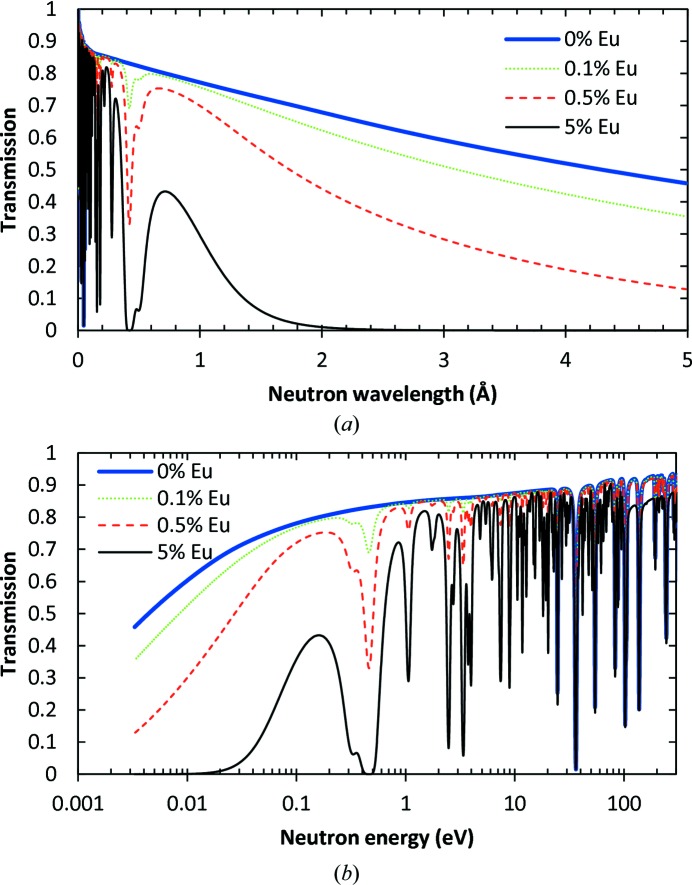
Neutron transmission spectra for BaBrCl:Eu samples with different concentrations of Eu dopant, as shown in the legend (in mole %), calculated from equations (2) and (3) using tabulated cross sections from the ENDF database. A constant 5 mm sample thickness, a natural isotopic abundance for Eu (^151^Eu 47.8% and ^153^Eu 52.2%) and a density of 4.5 Mg m^−3^ are assumed in the calculations. Strong neutron resonance absorptions at ∼0.42, 0.28, 0.22, 0.18 and 0.16 Å (∼0.46, 1.06, 1.76, 2.47 and 3.39 eV) are present for the Eu dopant and can be used for unambiguous imaging in energy-resolved experiments, as well as Ba and Br for high-energy resonances. In the thermal and cold neutron ranges, Eu provides nearly half of the attenuation contrast, even at the 0.5% dopant concentration.

The transmission of a sample at any position within the image (*x*, *y*) can be calculated from
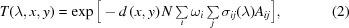
where *d* is the thickness of the sample at position (*x*, *y*), *N* is the number of molecules per unit volume, ω_*i*_ is the atomic fraction of the *i*th element, and σ_*ij*_(λ) and *A_ij_* are the neutron attenuation cross section for neutrons of wavelength λ for the *j*th isotope of element *i*, and the abundance of isotope *j* for element *i*, respectively. The number of molecules per unit volume can be obtained from the equation
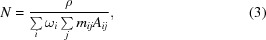
where ρ is the sample density and *m_ij_* is the atomic mass of isotope *j* of element *i*. The expected transmission of the BaBrCl:Eu sample was calculated from equations (2) and (3) for a 5 mm sample with various Eu doping levels: 0, 0.1, 0.5 and 5% (Fig. 4 ▸). The presence of many dips in the epithermal range of neutron energies is due to so-called resonance absorption (Lamb, 1939 ▸; Jackson & Lynn, 1962 ▸). The graphs in Fig. 4 ▸ demonstrate the possibility of performing element-specific imaging if neutron transmission spectra are measured in each pixel and the element of interest has a sufficiently large resonance attenuation cross section. Many of the dips in Fig. 4 ▸ are due to resonance absorption by Eu, while some of them are from resonance absorption by Ba and Br. The resonance absorption by Cl is negligible compared with the other elements in our samples. Element-specific imaging can be performed if only neutrons around the resonance energies are used to form an image, which is typically only possible at pulsed spallation neutron sources. In that case, the contrast in an image taken at specific resonance energies is dominated by the attenuation by that specific element (Schillebeeckx *et al.*, 2012 ▸; Kai *et al.*, 2013 ▸; Tremsin *et al.*, 2013*a* ▸,*b* ▸, 2014 ▸; Festa *et al.*, 2015 ▸) and this type of imaging was not available to us at the ICON and BOA facilities at the Paul Scherrer Institute.

It should also be noted that the neutron scattering cross section changes with temperature. The change in resonance absorption cross section due to Doppler broadening is not considered in this analysis, as our spectrum did not contain those energies. The neutron scattering cross section for thermal and cold neutron energies typically scales with temperature according to
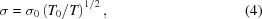
where σ_0_ is the tabulated scattering cross section at temperature *T*_0_. Therefore, the thermal and cold neutron scattering cross section changes by nearly 50% between room temperature and 1000 K, but only by ∼2% for the temperature change from 50 K below to 50 K above the melting point of 1159 K of BaBrCl, which was the range of temperatures used in the present melting/solidification imaging experiments. The observed changes in transmission are much larger than can be explained by a 2% change in scattering cross section due to temperature gradients. Thus, we rule out temperature dependence of the attenuation cross sections as the main reason for the observed changes, leaving the redistribution of elements (primarily the Eu dopant) as the main phenomenon to explain our results.

**Figure 5:**
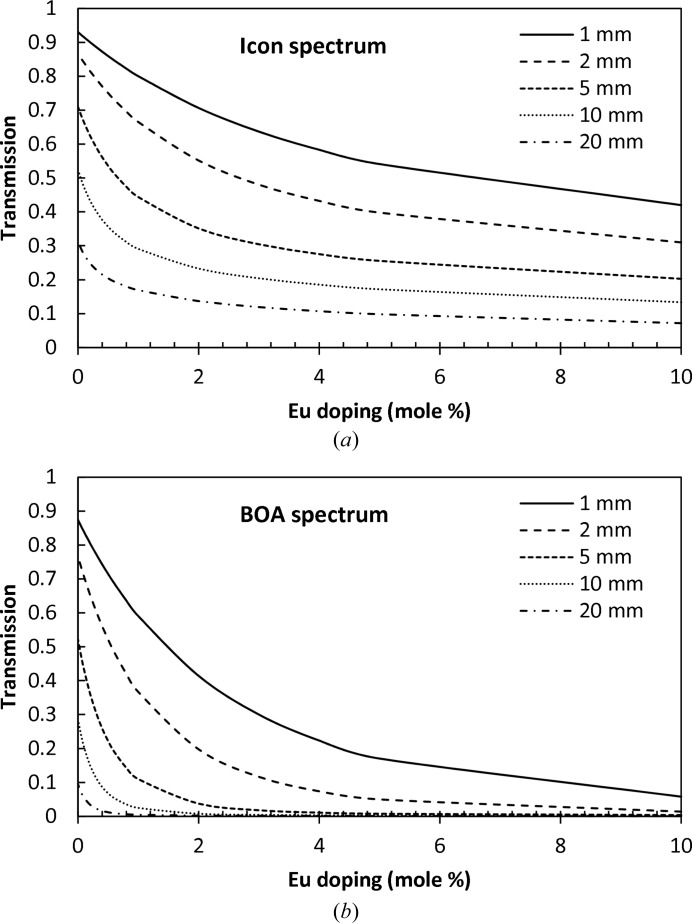
The theoretical transmission [calculated from equations (2) and (3)] of BaBrCl:Eu for the ICON and BOA neutron beam spectra as a function of Eu dopant concentration. The legend indicates the thickness of the material assumed in the calculation. The colder beam spectrum of the BOA beamline is more suitable for small dopant concentrations, while the ICON beam can be used for thicker samples.

The white-spectrum neutron imaging performed here has the advantage of using the full neutron flux available at continuous sources, in contrast with experiments where only a fraction of neutrons of particular wavelengths are selected by a particular monochromator device (*e.g.* a velocity selector or double-crystal monochromator; Wagner *et al.*, 1992 ▸; Treimer *et al.*, 2006 ▸; Schulz *et al.*, 2009 ▸). In the present experiments, the time-dependent sample transmission was recorded with an integration time of only 4–6 s and thus a full-beam flux option was chosen for the data acquisition. Thus, only one transmission value (namely the transmission value integrated over the entire beam spectrum) was obtained for each pixel of our data set. These integrated transmission values can be estimated from the equation
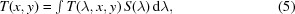
where *S*(λ) is the normalized neutron beam spectrum 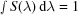. The two non-normalized spectra shown in Fig. 3 ▸ were used to calculate the expected BaBrCl:Eu transmission for samples of different thicknesses and different Eu dopant concentrations, as shown in Fig. 5 ▸. These theoretical predictions of sample transmission can be used for the spatially resolved reconstruction of the Eu concentration from the experimental data, provided that in each pixel (*x*, *y*) the neutron transmission and sample thickness can be measured accurately and the sample density is known. This last may change across the sample with the varying concentration of dopant, but can be reconstructed iteratively: after each iteration the sample density is corrected according to the reconstructed elemental composition and the calculation is repeated with new values for the density ρ(*x*, *y*).

Eu is one of the group of elements that have a relatively high neutron attenuation cross section in the thermal regime, which makes it possible to resolve its concentration to the level of a fraction of a percent, as seen from Fig. 5 ▸. In fact, for large Eu concentrations (above a few percent in our case) the BaBrCl:Eu scintillators become opaque to cold neutrons with wavelengths above ∼1.8 Å (Fig. 4 ▸). However, for a BaBrCl:Eu sample <10 mm thick, the uniformity of the Eu concentration can be studied on both cold and thermal neutron imaging beamlines with very good sensitivity for sub-percent Eu dopant concentrations. A thicker sample or a larger Eu concentration can be studied at other neutron facilities where both thermal and epithermal neutron fluxes are present, for example at pulsed neutron spallation sources where energy-resolved imaging can be performed in time-of-flight mode, enabling not only mapping of the concentration of the strongest attenuating element but also simultaneous mapping of multiple elements in one measurement (Schillebeeckx *et al.*, 2012 ▸; Kai *et al.*, 2013 ▸; Tremsin *et al.*, 2013*a* ▸,*b* ▸, 2014 ▸; Festa *et al.*, 2015 ▸).

### 3.2. Dynamics of Eu concentration during melting/solidification processes   

**Figure 6:**
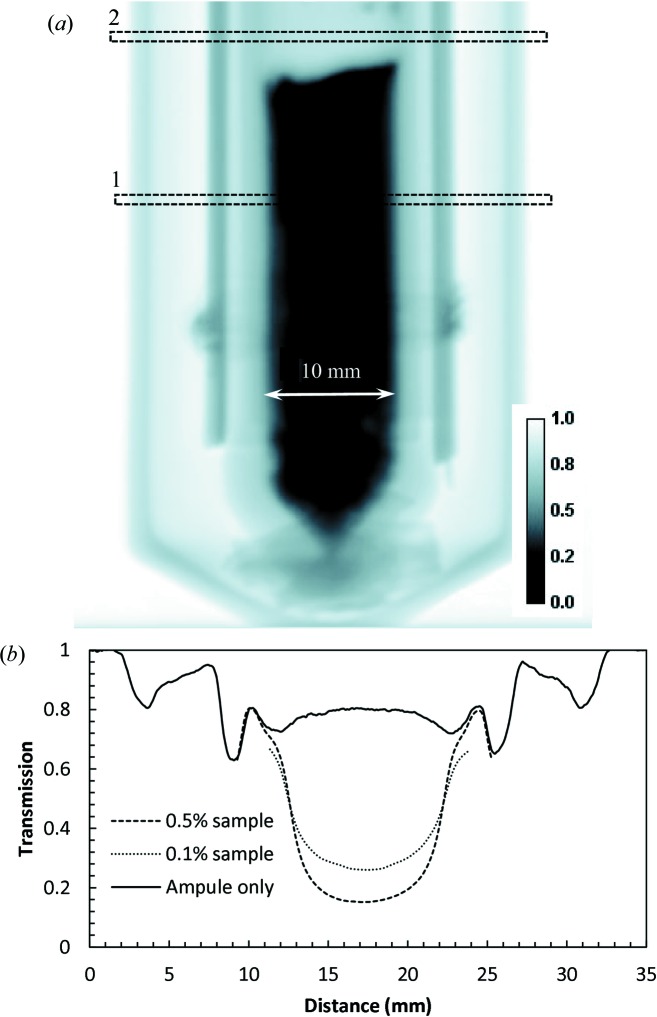
(*a*) A neutron transmission image of the BaBrCl:0.5%Eu sample with the heat absorbers mounted on the exterior of the sealed silica glass tube. BOA beamline, ∼2 min integration, original charge sample, measured at room temperature. (*b*) Cross sections through the image areas shown by the dashed rectangles.

A time-dependent study of Eu concentration during melting/solidification was performed on the BOA beamline with a BaBrCl:Eu sample mounted next to a lamp heater, as shown in Figs. 1 ▸ and 2 ▸. The neutron transmission image shown in Fig. 6 ▸ demonstrates that the transmission of the silica glass ampules, the NiO–YSZ heating radiation absorbers and the Alumel wires is sufficiently high to allow studies of the charge in the ampule. The combined attenuation of the heating radiation absorbers and two silica glass ampules was below 20% for the BOA beam spectrum, while the total transmissions in the thickest part of the sample were around 19 and 33% (corrected for the ampule transmission) (Fig. 6 ▸ *b*) for the samples with 0.5 and 0.1% Eu doping, respectively. The only difference between the preparation of these two samples is the different amount of Eu added to the original charge.

At this point we should clarify what determines the measured transmission in each pixel of our image. Obviously the sample thickness is one of the factors. The elemental composition is another factor and, among the four elements present in the charge, Eu has by far the largest attenuation cross section for thermal neutrons of 25 meV energy for the natural isotopic abundance (according to the databases of neutron scattering lengths and cross sections, *e.g.* that located at the NIST Center for Neutron Research at https://www.ncnr.nist.gov/resources/n-lengths/; see Table 1 ▸).

However, when the concentration of Eu is two orders of magnitude lower than those of Ba, Br and Cl, image contrast can be formed by the other elements if their distribution across the sample is non-uniform. In each particular material, the dependence of the measured transmission on the elemental composition can be evaluated from the tabulated energy-dependent cross sections (*e.g.* extracted from the ENDF database). In some cases, including the present study, prior knowledge of the sample materials can also be used to determine which elements might segregate into clusters, and it can be determined whether neutron imaging at a particular beamline can resolve them. Moreover, energy-resolved imaging over a wide range of energies, including the epithermal energies, can unambiguously map some elements independently from each other through resonance absorption imaging, as demonstrated previously (Schillebeeckx *et al.*, 2012 ▸; Kai *et al.*, 2013 ▸; Tremsin *et al.*, 2013*a* ▸,*b* ▸, 2014 ▸; Festa *et al.*, 2015 ▸). In our experiments with a white-beam thermal/cold spectrum, we determined that most of the contrast in the images of Eu-doped samples must be produced by the variation in Eu concentration, even at doping levels as low as 0.1%. The previous extensive research on BaBrCl has indicated that BaBrCl crystals grow as a solid solution of BaBr_2_ within BaCl_2_, and according to their phase diagram (Yan *et al.*, 2016 ▸) these cannot segregate into separate clusters. The uniformity of Eu doping is well known to be affected by multiple factors as its segregation coefficient is less than 1. The results presented below, where the transmission of BaBrCl:0.1%Eu changes from 0.52 to 0.36 within the center of the sample (∼10 mm thick section, ICON beamline spectrum), can easily be explained by the non-uniformity of the Eu concentration, expected to occur under rapid solidification. If the Eu concentration remained constant, the observed variation in image contrast would require a variation in the Cl concentration from 25 to 55% (with a nominal average of 33%), assuming Cl atoms are mutually replaced by Br atoms within the observed clusters, which is unlikely owing to the high solubility of BaBr_2_ within BaCl_2_. Moreover, energy-resolved neutron imaging of BaBrCl:Eu samples has indicated that the distributions of Ba and Br within the samples remain uniform, while Eu has a strong tendency to segregate during rapid solidification. Thus, we conclude that most of the contrast in our images is related to neutron attenuation by Eu, enabling study of the Eu concentration within the sample during the solidification and melting processes.

The melting and solidification were performed on a much faster time scale (∼minutes) compared with the normal crystal-growth procedure performed over timescales of hours to weeks. The intention here was to demonstrate that neutron imaging can be used as an *in situ* remote sensing tool providing direct information on the materials within the growth chamber, and not to optimize the crystal growth yet.

**Figure 7:**
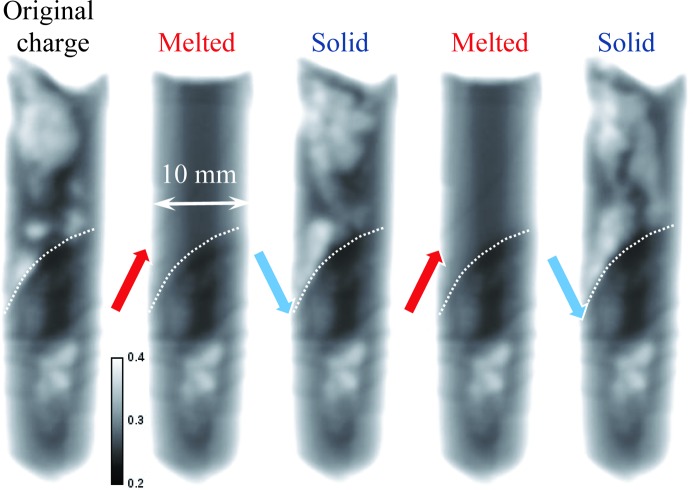
Neutron transmission images of the BaBrCl:0.1% Eu sample measured at the end of the heating/cooling cycles, when no apparent changes in the sample were observed. The arrows indicate whether the sample was cooled or heated between two consecutive images. The bottom part of the sample was never melted and remained in the original charge condition. Most of the contrast in these images is due to Eu attenuation of the cold neutrons of the BOA beamline facility. The dashed lines indicate the approximate position of the interface between the melted and original charge sections of the sample.

**Figure 8:**
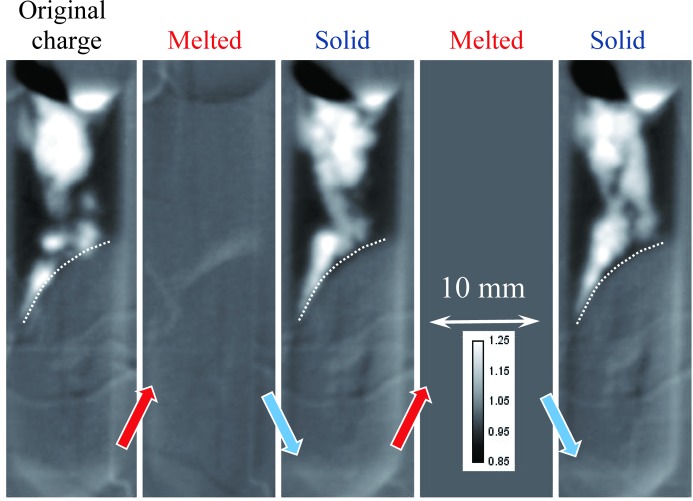
The same as Fig. 7 ▸, except normalized by the second melted state. The dependence on sample thickness is eliminated by this normalization. Only differences between the solid and melted states are seen in these images. The lighter areas correspond to Eu deficiency, while darker areas show increased Eu concentration compared with the melted state. The dashed lines indicate the approximate position of the interface between the melted and original charge sections of the sample.

**Figure 9:**
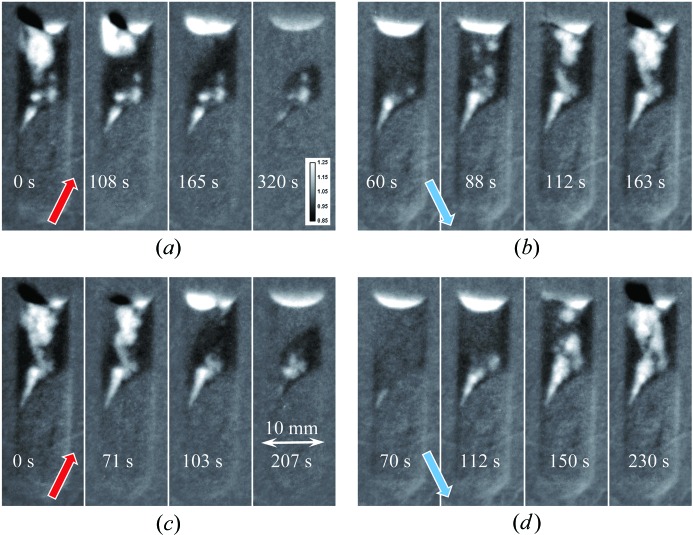
Neutron transmission images measured at different times within heating/cooling cycles normalized by the image acquired in the melted state. (*a*), (*c*) Heating cycle, with the temperature set to ∼1273 K (∼50 K above the melting point of the BaBrCl material). (*b*), (*d*) Cooling cycle, with the temperature maintained at ∼1173 K. The legend in each image indicates the time since the start of the corresponding cycle.

Several melting–solidification cycles were repeated for our two samples in order to investigate the randomness of the elemental distribution within the samples. The bottom part of each sample remained in as-prepared condition (the area separated by a dashed line in Figs. 7 ▸ and 8 ▸). The heat source, and therefore the highest temperature, is on the left-hand side of Figs. 7 ▸–11 ▸ ▸ ▸ ▸. The first image of Fig. 7 ▸ indicates the presence of a strong Eu concentration variation in the as-prepared sample (polycrystalline charge), which completely disappeared in its upper section once the sample was melted. The structure of the re-solidified material was globally similar to the initial charge, although the segregation of Eu atoms was quite random within the sample volume and appeared to form clusters on multiple scales, as revealed by our tomographic measurement reported in §3.3. The temperature of the sample was kept at ∼50 K above or below the melting point of 1159 K for the melting and solidification cycles, respectively. The same data set normalized by the second melted state, shown in Fig. 8 ▸, emphasizes the changes in Eu concentration. The boundary between melted and solid material can also be clearly seen in these images, as no variation in Eu concentration is observed within the initial charge section of the sample. A higher concentration of Eu at the top left location can probably be explained by the fact that this area solidified last and Eu was expelled towards there. This hypothesis is supported by the individual images taken during the melting and solidification processes, shown in Fig. 9 ▸ and the supporting information, where the dynamics of the elemental distribution are visualized in a time-lapse sequence. It is seen from these images that, during solidification, Eu-deficient clusters first formed close to the solid interface at the bottom of the sample, as well as in the very top area, then larger clusters were formed and eventually the remaining liquid-phase Eu was expelled all the way to the top of the sample. The middle section of the sample solidified into the compound with reduced Eu concentration. In the melted state, the Eu distribution within the sample’s liquid volume remained quite uniform. The same general pattern persisted over repeated cycles. Upon reduction of the heater power (to ∼50 K below the melting point), the solidification proceeded rapidly and simultaneously from the solid–liquid interface and for the top of the melt on the side opposite the heater. This strong non-uniformity of the temperature field explains the complex (even turbulent) pattern observed in the Eu segregation. The reproducibility of the observed dopant segregation indicates that the Eu concentration is highly dependent on the local temperature uniformity during the solidification process, while at the same time the Eu mobility is high enough to adapt to these differences in temperature within the melted state.

**Figure 10:**
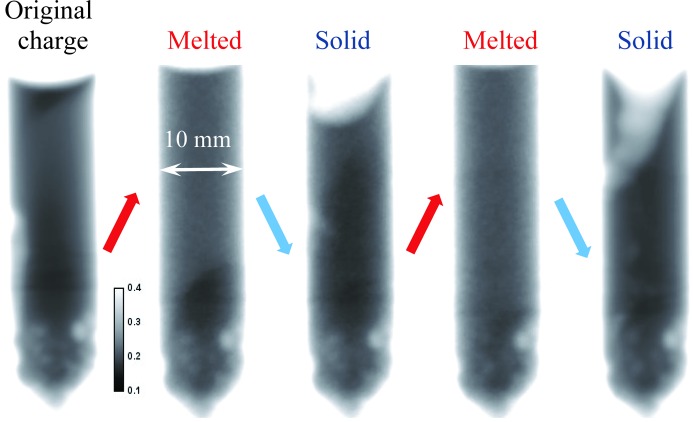
The same as Fig. 7 ▸, except measured for the BaBrCl:0.5% Eu sample.

Very similar results were obtained with the BaBrCl:0.5%Eu sample, as shown in Figs. 10 ▸ and 11 ▸, except for the fact that this sample had a more uniform distribution of Eu in the charge as well as in subsequent melted/solidified conditions. It could be inferred that the strong clustering of the Eu is more related to the homogenization time of the charge than to the concentration. No Eu-rich areas were observed at the top of this sample in its re-solidified state, opposite to the previous case with 0.1% concentration. Most of the Eu-deficient areas of the sample were located at its top section in the solid state after the initial melting (initially the as-received state showed increased Eu concentration in the top section), with a few small clusters seen in various locations of the re-solidified section of the sample. The differences observed between these two samples with 0.1 and 0.5% Eu doping are likely related to the original charge uniformity, as subsequent rapid melting/solidification in the neutron beam exhibited the same difference in the uniformity of the Eu distribution within the samples. The charge with 0.5% Eu doping was homogenized for an extra hour, directionally solidified and cooled at a rate of 40 K h^−1^, while the charge with 0.1% Eu doping was more rapidly homogenized and cooled. This resulted in a more homogeneous charge for the 0.5% Eu-doped sample (but still not as good as it would be for a standard single-crystal growth procedure). However, these differences should not be extrapolated to properly grown γ-ray scintillator crystals, as the intention of this study was to demonstrate the possibility of *in situ* crystal diagnostics, rather than to characterize the crystals themselves.

### 3.3. Three-dimensional distribution of Eu dopant within the BaBrCl:0.1%Eu sample   

**Figure 12:**
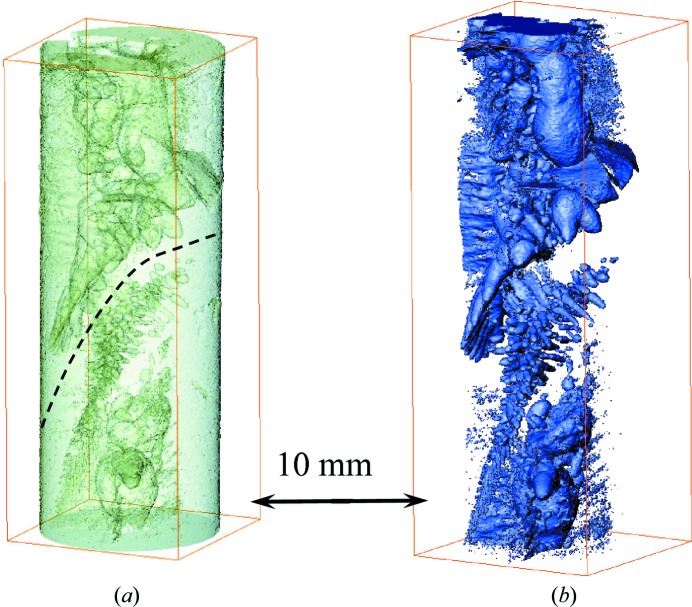
A tomographic reconstruction of the BaBrCl:0.1% Eu sample measured after the last cooling cycle. The ‘bubbles’ observed inside the sample are areas which are Eu-deficient compared with neighboring areas. (*a*) Both the bulk of the sample material and the Eu-deficient areas are shown. (*b*) Only the Eu-deficient areas are selected in this reconstruction. The dashed line shows the approximate location of the interface between the original charge and melted/re-solidified parts of the sample. The tomographic data set was measured on the ICON beamline.

To study the distribution of the Eu concentration in more detail, the same BaBrCl:0.1%Eu sample was measured in a tomographic mode on the ICON cold neutron beamline (Kaestner *et al.*, 2011 ▸) after the last solidification process. The last transmission images of Figs. 7 ▸, 8 ▸ and 9 ▸ correspond to the same sample conditions as the one shown in Fig. 12 ▸ after tomographic reconstruction from 376 projections taken over 360° sample rotation with 60 s image acquisition per projection. The longer image acquisition and the more optimal setup geometry in this measurement, namely a much shorter distance between sample and detector, resulted in improved spatial resolution and overall image quality. Indeed, the heating and cooling equipment used in the *in situ* melting experiments on the BOA beamline required a separation of ∼15 cm between the sample and the detector active area, while on the ICON beamline the sample was mounted few millimetres away from the detector. The finite neutron beam divergence resulted in some image blurring of our melting experiments, reported in the previous section. The results of the tomographic reconstruction in Fig. 12 ▸ indicate that Eu-deficient clusters exist in the entire sample volume, both in the charge area and in the melted/re-solidified area. The sample imaged in tomographic mode was not solidified in a directionally controlled manner at a slow rate, as in the case of good quality single-crystal γ-ray scintillators. The last solidification was performed with a highly inhomogeneous temperature profile and lasted only several minutes, which led to the formation of Eu-deficient clusters. The size of the observed clusters spans a relatively large length scale, as can be seen in Fig. 12 ▸. The interface area between the charge and melted volumes seems to lack these clusters, or they are below our spatial resolution. As segregation occurs during crystal growth of doped materials, this study demonstrates that *in situ* imaging can be used to evaluate its extent and to provide real-time feedback to be used for process optimization.

### 3.4. Correlation of sample cracking with the uniformity of elemental composition   

**Figure 13:**
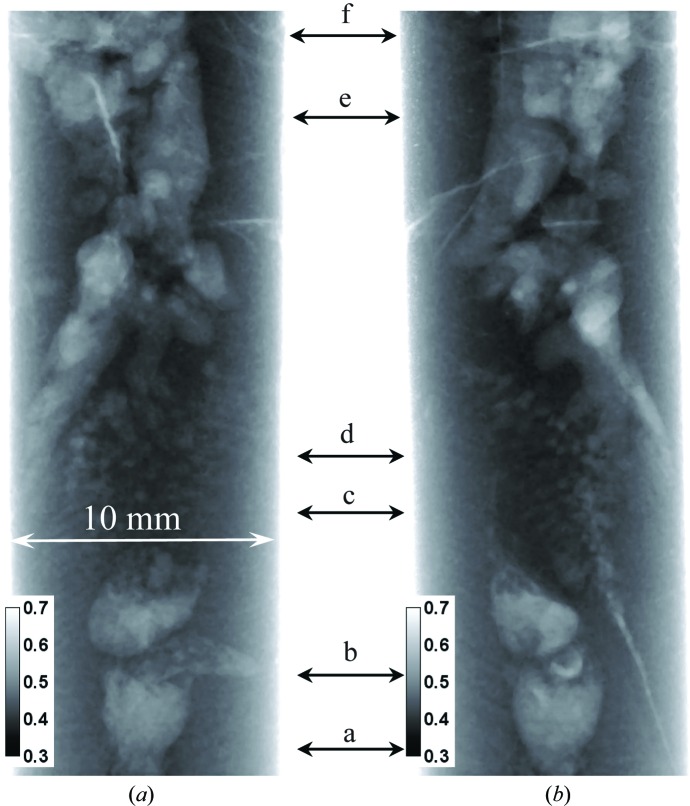
Neutron transmission images of the BaBrCl:0.1%Eu sample measured after the last cooling cycle. Two nearly orthogonal sample orientations are shown in images (*a*) and (*b*). Data recorded on the ICON beamline. Multiple cracks are seen in the images, as well as areas of reduced Eu concentration. The arrows with letters indicate the positions of the horizontal slices through the tomographic reconstruction shown in Fig. 15.

**Figure 14:**
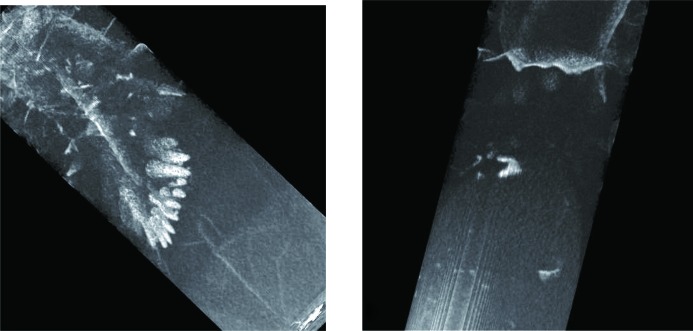
Tomographic reconstructions of the BaBrCl:0.1%Eu sample revealing the cracks, with their location correlated with the locations of Eu deficiency. More details of the internal crack locations are shown in Fig. 15. Two sample orientations are shown, with cracks propagating deep into the sample.

**Figure 15:**
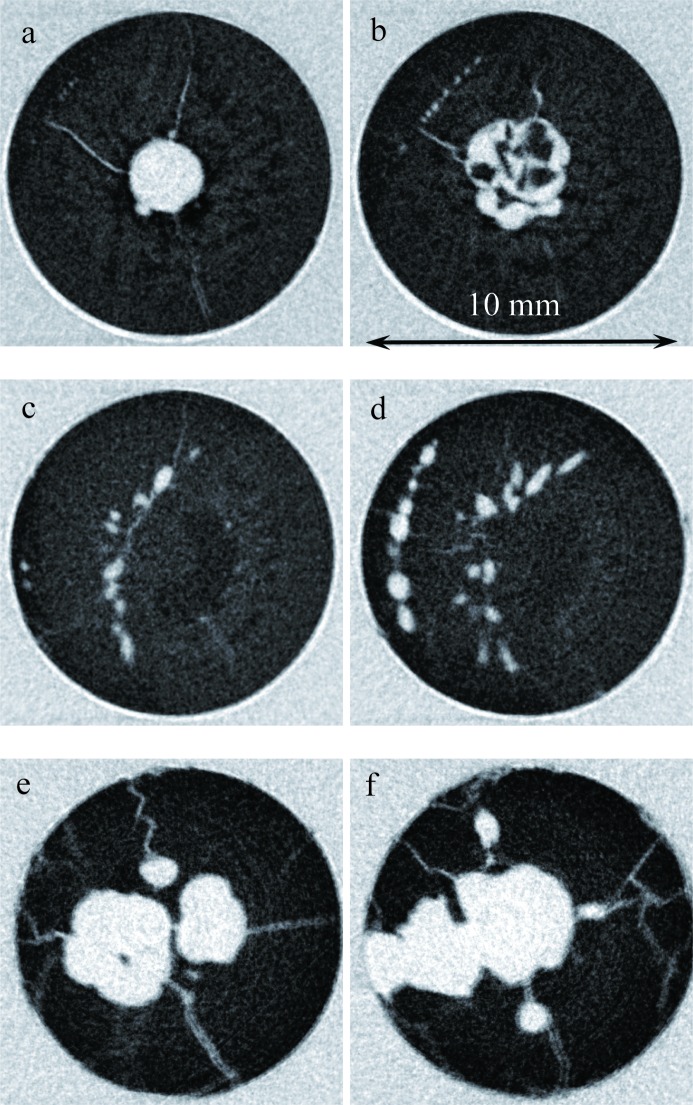
Slices through the tomographic reconstruction of the BaBrCl:0.1%Eu sample. The letters in each part are the locations of the slices within the sample as indicated in Fig. 13 ▸. All the cracks observed in that sample originate within an Eu-deficient volume.

It was noticed in our transmission images that multiple cracks existed in the sample through the entire volume and that most of them were spatially correlated with the location of Eu-deficient areas. The two orthogonal projections shown in Fig. 13 ▸ demonstrate that some cracks can be seen even in single projection images, which can be taken *in situ* as the crystals are grown. However, more detailed information on crack location can be obtained in a tomographic reconstruction, which can in principle be done *in situ* as well, but which requires longer data integration (not a major issue, since typical crystal-growth processes are very slow). The extent of these cracks is obviously seen better in the tomographic reconstructions, as shown in Figs. 14 ▸ and 15 ▸. The cracks within the sample originate in an area where the Eu concentration is reduced compared with the neighboring volume. Many of them propagate from the sample core with reduced Eu concentration towards the sample periphery, along the radial direction. The panels labeled c and d in Fig. 15 ▸ confirm our previous observation that, in the interface area between the charge and melted regions, the distribution of Eu is more uniform and no large clusters with changed Eu concentration are present. Most likely, these cracks have formed as a result of a relatively fast sample-cooling process, much faster than in real crystal-growth processes, leading to thermal stresses, which result in cracks. However, the fact that the cracks originate from areas with reduced Eu concentration may still be relevant for the production of real γ-ray scintillation materials. If confirmed, this would indicate that process optimization can be directed towards crystal growth with an emphasis on the uniformity of the dopant distribution, which in turn is related to the shape of the solid/liquid interface and the overall thermal environment. Neutron imaging can be very helpful for this optimization by providing *in situ* mapping of elemental concentration.

### 3.5. Quantification of Eu concentration within the sample   

In general, our neutron imaging experiments performed with a white-beam spectrum do not provide full information on the elemental distribution within the sample. However, in the particular case of BaBrCl:Eu, where thermal and cold neutron attenuation is dominated by a single element (namely Eu, as seen in Fig. 4 ▸), the distribution of that element can still be studied with some quantification. The Eu concentration parameter ω_Eu_ can in theory be obtained from the measured sample transmission *T*(*x*, *y*) using an integral equation derived from equations (2), (3) and (5),
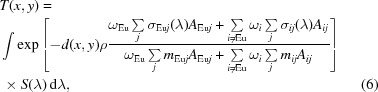
provided the sample thickness *d*(*x*, *y*) is known in all pixels (*x*, *y*). In our data analysis we used a simplified numerical approach, based on the calculated transmission spectra from equations (3) and (5) (similar to those shown in Fig. 4 ▸) and the measured beam spectrum of the ICON beamline shown in Fig. 3 ▸. First, the function inverse to the calculated sample transmission shown in Fig. 5 ▸(*a*) was approximated by a polynomial:
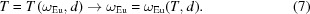
This inverse function was then used to recover the parameter ω_Eu_ for each pixel of our measured data. The known cylindrical geometry of the sample was used to calculate the sample thickness *d*(*x*, *y*) = (*R* ^2^ − *x* ^2^)^1/2^, with *x* = 0 assumed to be in the center of the sample and *R* the radius of the sample measured to be ∼5 mm.

**Figure 16:**
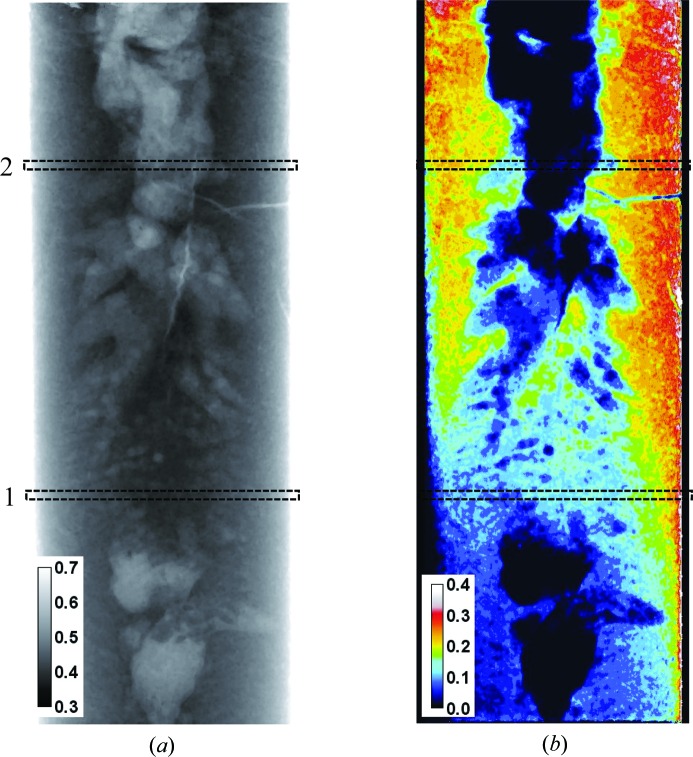
(*a*) A neutron transmission image of the BaBrCl:0.1%Eu sample sealed in a silica glass ampule, normalized by the ampule transmission. The contrast in this image is determined by both elemental composition and sample thickness. Data recorded on the ICON beamline. The legend indicates measured transmission values. The dashed rectangles indicate the areas used for the cross sections through the images shown in Fig. 17 ▸. (*b*) A map of the reconstructed Eu concentration within the sample. The legend indicates the Eu doping level in mole %.

The map of Eu concentration reconstructed for the projection shown in Fig. 16 ▸(*a*) demonstrates the sensitivity of this method to a sub-percent Eu concentration in BaBrCl samples of 1 cm thickness. The accuracy of our reconstruction depends on the precision of the measured neutron transmission values, which in turn depends on the quality of the neutron beam (background, γ-ray contamination, collimation and beam stability), the neutron imaging detector, neutron counting statistics and neutron scattering in the sample. This experiment was neither optimized for a very accurate quantitative measurement of sample transmission nor in general intended for an accurate quantification of Eu concentration within the sample. Therefore, we omit here a detailed analysis of the ultimate sensitivity limit of this method and can only state that it is likely that this technique can reconstruct Eu concentration in BaBrCl:Eu samples to sub-0.1% level, as shown in Figs. 16 ▸ and 17 ▸. However, the sum of measured concentration across the entire Eu distribution map shown in Fig. 16 ▸(*b*) can be used for self-consistency, as the integrated Eu concentration should agree with the value of 0.1% in the material charge. The average concentration of Eu shown in Fig. 16 ▸(*b*) is found to be 0.113%. The edges of the reconstructed map (where the sample thickness changes very rapidly) have the largest error in the reconstructed concentration due to the largest error in the sample thickness parameter used in the reconstruction. Moreover, this is not a generally applicable method, since not all materials can be studied in the white-beam experiment described in this paper, and only sample compositions where most of the imaging contrast is provided by a single element/molecule can be treated in a similar way. However, similar measurements performed with good neutron energy resolution, where transmission curves similar to the predicted curves of Fig. 4 ▸ can be obtained, will allow remote *in situ* simultaneous quantification of elemental composition for multiple materials.

## 4. Conclusion   

The neutron imaging experiments conducted at the Paul Scherrer Institute have demonstrated the possibility of remote study of several properties of scintillator materials, which can be conducted *in situ* while the crystals are grown. The thermal and cold neutrons used for this imaging have low attenuation for various materials used in typical furnaces (*e.g.* various metals, insulation ceramics), which are opaque to other more conventional remote imaging techniques, *e.g.* based on X-ray photons. Some internal microstructure characteristics of γ-ray scintillator crystals, such as the uniformity of their elemental composition, or the presence of cracks and voids, can be studied in real time, considering the fact that the growth processes are relatively slow. Thus, it is demonstrated that the *in situ* dynamics of elemental composition can be investigated by neutron imaging for some materials.

The interface between the solid and liquid phases can be imaged in some material compositions which have a relatively high neutron attenuation coefficient for an element exhibiting a measurable increase in its concentration across the interface (*e.g.* dopants which tend to have a larger concentration in the liquid phase). Our study of the Eu concentration in BaBrCl:Eu samples revealed the strong tendency of Eu to segregate into clusters during material solidification, repeated over several cycles. Clusters with lower Eu concentrations were observed to form preferentially in the core of our sample, with cluster sizes spanning large length scales (<0.1–5 mm). It was also observed that crystal cracking was spatially correlated with those clusters with reduced Eu concentration. We have also demonstrated that some quantification of elemental composition can be performed for systems where the neutron attenuation is dominated by a particular element, as demonstrated by the analysis of our transmission imaging combined with the theoretical calculation of transmission based on tabulated neutron cross-section data.

In summary, neutron imaging can be an attractive technique for the optimization of parameters for a particular crystal-growth method by providing real-time information on the material properties while samples are in a process chamber. Extension of the described imaging techniques to energy-resolved neutron imaging can substantially improve the accuracy of the reconstructed elemental composition, which can be done for multiple materials in one measurement. In particular, element-specific imaging (Schillebeeckx *et al.*, 2012 ▸; Kai *et al.*, 2013 ▸; Tremsin *et al.*, 2013*a* ▸,*b* ▸, 2014 ▸; Festa *et al.*, 2015 ▸) and *in situ* temperature measurement through Doppler broadening of resonances (Mayers *et al.*, 1989 ▸; Stone *et al.*, 2005 ▸; Yuan *et al.*, 2005 ▸; Tremsin, Kockelmann *et al.*, 2015 ▸) can be performed in such experiments. The method can also allow studies of such crystal properties as very small angular mis­alignments within the crystal, and the presence of grains, texture and possibly strain, from the transmission diffraction imaging. The techniques described here are not likely to be implemented in very many crystal-growing facilities as they require intense collimated neutron fluxes, which are presently only available at large-scale neutron facilities, but the optimization of the growth process can be done at these facilities and subsequently transferred to production units.

## Supplementary Material

Movies demonstrating the dynamics of the elemental distribution during melting and solidification. DOI: 10.1107/S1600576716004350/ks5504sup1.zip

## Figures and Tables

**Figure 11:**
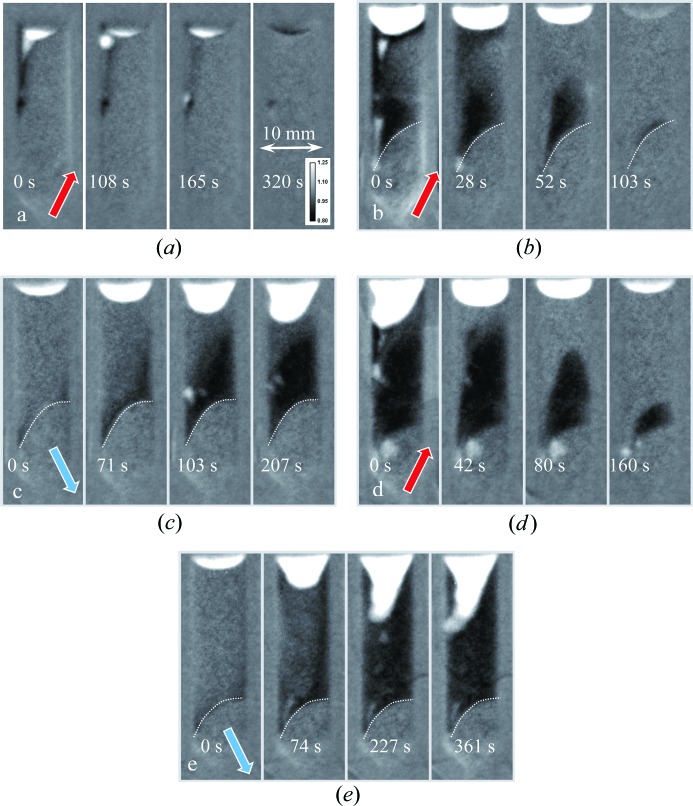
The same as Fig. 9 ▸, except measured for the BaBrCl:0.5% Eu sample. The sample was solidified between the first two heating cycles, with no data recorded during that process. The dashed lines indicate the approximate position of the interface between the melted and original charge sections of the sample.

**Figure 17:**
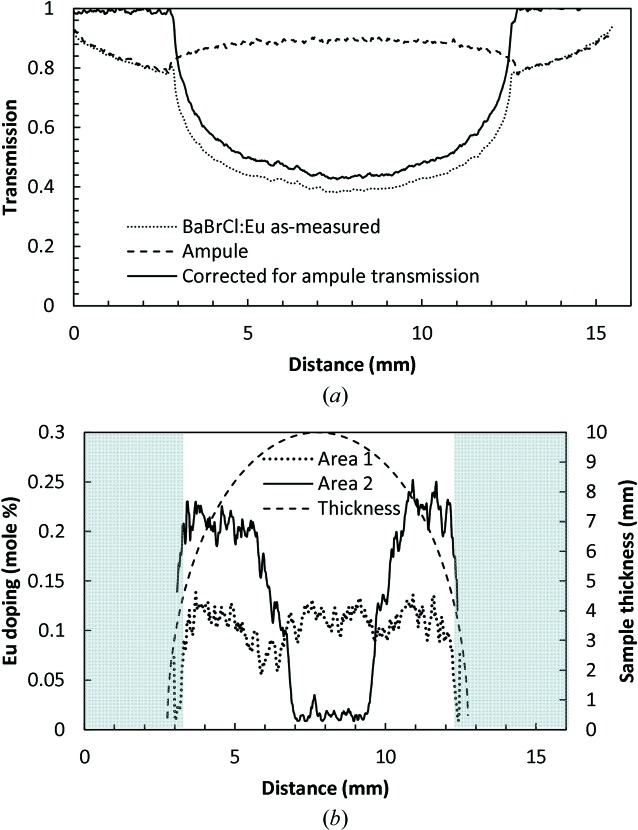
(*a*) The cross section through area 1 of Fig. 16 ▸(*a*). The measured transmissions for the sample and for an empty silica glass ampule are shown by dashed curves. The solid curve shows the transmission of the BaBrCl:0.1%Eu sample corrected for the ampule transmission. (*b*) Cross sections through the reconstructed map of Eu concentration of Fig. 16 ▸(*b*). The Eu concentration cannot be reconstructed accurately at the edges of the sample (shaded areas), where its apparent thickness seen by the neutron beam changes very rapidly (indicated by the dashed curved line).

**Table 1:** Neutron cross sections for the elements in this study 1 barn = 10^−28^ m^2^.

Element	σ_abs_ (barn)	σ_scatt_ (barn)
Ba	1.1	3.38
Br	6.9	5.9
Cl	33.5	16.8
Eu	4530	9.2
