# Supplementary material for: In situ diagnostics of the crystal-growth process through neutron imaging: application to scintillators
Source: J Appl Crystallogr. 2016 Apr 12;49(Pt 3):743–55. doi: 10.1107/S1600576716004350 (PMC4886976; doi:10.1107/S1600576716004350)
Supplement: Supplementary file 1 [file j-49-00743-sup1.zip › SupplementaryData.docx]

01_0.5%Sample_Heating.avi

0.5% Eu doping sample. Heating from room temperature to ~850 C. Ratio to the image acquired at the melted state (end of the movie) is shown. The Darker areas correspond to reduced Eu concentration, compared to the melted state, white areas have more Eu than in the melted state. In real time that sequence was approximately 14 minutes.

02_0.5%Sample_Cooling.avi

0.5% Eu doping sample. Cooling from ~850 C to room temperature. Ratio to the image acquired at the melted state (end of the movie) is shown. The Darker areas correspond to reduced Eu concentration, compared to the melted state, white areas have more Eu than in the melted state. In real time that sequence was approximately 8 minutes.

03_0.1%Sample_Heating.avi

0.1% Eu doping sample. Heating from room temperature to ~850 C. Ratio to the image acquired at the melted state (end of the movie) is shown. The Darker areas correspond to reduced Eu concentration, compared to the melted state, white areas have more Eu than in the melted state. In real time that sequence was approximately 11 minutes.

04_0.1%Sample_Cooling.avi

0.1% Eu doping sample. Cooling from ~850 C to room temperature. Ratio to the image acquired at the melted state (end of the movie) is shown. The Darker areas correspond to reduced Eu concentration, compared to the melted state, white areas have more Eu than in the melted state. In real time that sequence was approximately 7 minutes.

05_0.1%Sample_Tomographic1.mp4, 06_0.1%Sample_Tomographic1.mp4

Tomographic reconstruction of sample with 0.1% Eu doping. The darker areas correspond to Eu deficiency or cracks. Most of the cracks originate at the Eu-deficient areas.
